# Predicting complications in emergency department patients with acute coronary syndrome – Existing risk scores versus a new logistic regression model

**DOI:** 10.1016/j.ahjo.2026.100736

**Published:** 2026-02-10

**Authors:** T. Nilsson, M. Strömfors, A. Trägårdh, A. Mokhtari, A.M. Khoshnood, U. Ekelund

**Affiliations:** a Emergency Medicine, Department of Clinical Sciences, Lund University, Lund, Sweden; b Department of Emergency Medicine, Skåne University Hospital, Lund, Sweden; c Department of Cardiology, Skåne University Hospital, Lund, Sweden; d Emergency Medicine, Department of Clinical Sciences, Lund University, Malmö, Sweden; e Department of Emergency Medicine, Skåne University Hospital, Malmö, Sweden

**Keywords:** Acute coronary syndrome, Emergency department, Complications, Predictors, Risk stratification

## Abstract

**Background:**

Patients with acute coronary syndrome (ACS) are often admitted to monitored wards due to the risk of complications. Several risk prediction scores exist, but their use in the emergency department (ED) is limited. We aimed to compare the ability of existing risk scores with a new logistic regression model in predicting complications in ACS patients.

**Methods:**

This was a secondary analysis of data from the ESC TROP trial (NCT03421873), including ACS patients from five EDs in Region Skåne, Sweden (2017–2018). Complications were identified via diagnosis and/or intervention codes and manual chart review. GRACE, GRACE FFE, TIMI, HEART, ACTION ICU, and CHA₂DS₂-VASc scores were calculated. A new logistic regression model was developed, and its predictive performance was assessed using the area under the ROC curve (AUROC) and a net reclassification improvement analysis (NRI).

**Results:**

Among 2223 ACS patients, 164 (7.4%) experienced complications. Independent predictors for complications included age, STEMI, troponin and lactate at arrival, shock index, Killip class, and new ECG changes. The logistic regression model's AUROC 0.84 (95% CI 0.80–0.88) outperformed all known risk scores: GRACE FFE 0.79 (0.75–0.84), ACTION ICU 0.77 (0.72–0.82), GRACE 0.76 (0.70–0.81), TIMI 0.74 (0.68–0.79), HEART 0.69 (0.64–0.74), and CHA₂DS₂-VASc 0.64 (0.59–0.69). Logistic regression improved reclassification of non-events, with a positive non-event NRI compared with all other scores.

**Conclusions:**

Serious complications occurred in 7% of ACS patients. A logistic regression model based on simple ED variables showed excellent predictive performance, surpassing existing risk scores. Improved risk stratification may optimize resource allocation while maintaining patient safety.

## Highlights

• Serious complications occurred in 7% of all ACS patients.
• Predictors of complications - age, STEMI, troponin and lactate at arrival, shock index, Killip class, and new ECG changes.
• A new logistic regression model demonstrated excellent predictive performance (AUROC 0.84) and outperformed six established risk scores.

## Graphical abstract

**Figure:**
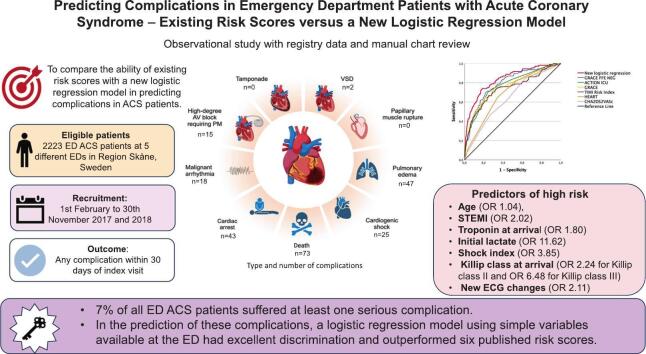

## 1 Introduction

Acute chest pain is a common symptom and accounts for around 5–10% of all emergency department (ED) visits annually [1]. A potentially life-threatening cause for acute chest pain is acute coronary syndrome (ACS) which includes ST-elevation myocardial infarction (STEMI), non-ST-elevation myocardial infarction (NSTEMI) and unstable angina (UA) [2]. Recent guidelines recommend that all ACS patients should be admitted to the coronary care unit (CCU), the intensive care unit (ICU) or wards where cardiac monitoring is available due to the risk of severe complications such as malignant arrhythmias, acute heart failure, and/or death [3]. However, while these complications are relatively uncommon, high-level admissions contribute to high costs and often there is shortage of beds [4]. A recent study found that ACS patients occupy up to 56% of CCU beds, yet only 5% of these patients required care beyond telemetry [5]. With advances in modern care, there is no firm evidence that all ACS patients require high-level admission [6]. Improved risk stratification at the time of admission could help distinguish truly high-risk patients from those who may safely receive lower levels of care, allowing for more efficient resource allocation while maintaining patient safety.

There are several published scores which may be used to predict the risk of complications in ED ACS patients, such as the Global Registry of Acute Coronary Events (GRACE) or the Acute Coronary Treatment and Intervention Outcomes Network (ACTION) ICU scores [7], [8]. However, their accuracy remains uncertain, and their complexity limits widespread use in the ED. There is thus a great need for more practical and reliable risk stratification models to support clinical decision-making. In the present study, we aimed to compare the ability to predict complications of existing risk scores in ED ACS patients with that of a newly developed logistic regression model.

## 2 Methods

### 2.1 Study design

This study is a secondary analysis of data from the ESC-TROP trial (NCT03421873) - a multicenter study that included consecutive ED patients aged 18 years and above with a main complaint of non-traumatic chest pain and at least one high-sensitive cardiac Troponin T (hs-cTnT) analyzed [9]. Patients were included from five EDs in Region Skåne, southern Sweden (Lund, Malmö, Helsingborg, Kristianstad and Ystad) from February 1st to November 30th in both 2017 and 2018. All patients were enrolled by default and informed via leaflets and posters at the EDs that they could decline participation at any time and for no reason. STEMI patients diagnosed in the ambulance were taken directly to the catheterization lab or the CCU, bypassing the ED, and were therefore not included in the study. Patients with missing data, a chief complaint other than chest pain, or a final diagnosis other than ACS following manual chart review were excluded from the study.

The study was approved by the regional ethics review board in Lund, Sweden.

### 2.2 Data collection and outcomes

The ESC-TROP database integrates data from all regional electronic patient records, along with the SWEDEHEART quality register, the Swedish emergency care register (SVAR) and the Swedish national patient register [10], [11], [12]. Patients with index-visit ACS diagnoses were identified using predetermined ICD-10 and intervention (KVÅ) codes. A comprehensive manual review of all regional patient records was then performed. Three authors (TN, MS, and AT), supported by research assistants, conducted the review, collecting data on complications, admission ward, treatment strategy, vital signs, blood tests, and risk factors such as smoking and family history of cardiovascular disease. For quality assurance, TN reviewed the records of all patients.

In the primary database search, ACS was defined as a primary or secondary diagnosis at the index-visit with the ICD-10 codes I200, I21, I22, I248 and I249. In the manual chart review, acute myocardial infarction (AMI) was defined according to the universal definition and required signs or symptoms of acute ischemia in combination with a significant increase and/or decrease in hs-cTnT with at least one value above the 99th percentile [13]. In case of late presentation to the ED, patients with elevated hs-cTnT and no significant rise or fall could still be diagnosed with AMI, if this was deemed to be the most likely diagnosis. A UA diagnosis was made in the setting of normal or only slightly elevated hs-cTnT levels (from e.g., heart or kidney failure) without a significant increase and/or decrease [14] and a symptom history consistent with UA.

Complications were identified using predetermined ICD-10 and intervention (KVÅ) codes along with manual chart review, and were defined as the following events: death; cardiac arrest where cardio-pulmonary resuscitation was provided; cardiogenic shock; pulmonary edema requiring mechanical ventilation (non-invasive or invasive); ventricular tachycardia where antiarrhythmic treatment or electrical cardioversion was provided; high-degree AV-block requiring a pacemaker (PM) implantation; PM or implantable cardioverter-defibrillator (ICD) implantation; implantation of a circulatory assist device, such as an intra-aortic balloon pump (IABP), a percutaneous ventricular assist device (Impella pump), or extracorporeal membrane oxygenation (ECMO); and mechanical complications, including cardiac tamponade due to left ventricular free wall rupture, papillary muscle rupture, or ventricular septum defects (VSD). These complications were chosen based on clinical judgment and reflect events resulting from ACS that frequently require admission to the CCU/ICU as well as the use of advanced monitoring and therapeutic interventions.

Patients with complications before hospital arrival or at the ED, as well as hemodynamically unstable patients (Killip class IV) are known to be at high risk and are justifiably admitted to the CCU/ICU. These patients were therefore excluded in the final analysis, as were patients with palliative care from the ED. We also excluded complications strictly due to invasive procedures, such as coronary angiography or coronary artery bypass graft surgery (CABG), as these complications are likely hard to predict from the ED.

Treatment strategy was defined as invasive if coronary angiography or other invasive procedures (direct CABG if known coronary anatomy) were performed, conservative if only medical ACS treatment was administered, and palliative when only comfort care was given. The interpretation of electrocardiograms (ECGs) was performed manually by TN in accordance with current guidelines [3]. New ischemic ECG changes were defined as new significant ST-depression or elevation, T-wave abnormalities, or pathological Q-waves, as well as new left or right bundle branch block compared to a prior ECG.

### 2.3 Risk prediction models

Based on data from the manual chart review, we calculated the following six risk scores in each ACS patient: Thrombolysis in Myocardial Infarction (TIMI) risk index; Global Registry of Acute Coronary Events (GRACE), GRACE freedom from events (GRACE FFE); History, ECG, Age, Risk Factors and Troponin (HEART); Acute Coronary Treatment and Intervention Outcomes Network (ACTION) ICU and CHA₂DS₂-VASc.

GRACE [7], TIMI risk index [15], and HEART [16], [17] predict mortality and higher values indicate greater risk. The GRACE Freedom from event (FFE) score is based on GRACE and predicts the likelihood of remaining free from adverse in-hospital events in patients with NSTEMI or UA [18]. A high GRACE FFE indicates a low risk of adverse events. The calculation of both GRACE and GRACE FFE scores usually require software due to their complexity. The ACTION ICU score is designed to predict the need for ICU care in initially stable NSTEMI patients [8]. The CHA₂DS₂-VASc score was developed to determine the need for anticoagulants in patients with atrial fibrillation. Studies suggest that CHA₂DS₂-VASc may also effectively predict in-hospital mortality and major adverse cardiac events (MACE) in patients with ACS [19], [20].

If smoking status or family history data were missing when calculating the scores, we assumed that the patient was a non-smoker and had no family history of cardiovascular disease. However, if data for other essential variables required for score calculation were missing, the scores were not calculated.

### 2.4 Statistical analyses

We used frequencies and percentages to describe categorical data. For continuous data we used mean and standard deviation (SD) for normally distributed data and median and interquartile ranges (IQR) for skewed data. Due to skewed distributions, hs-cTnT and lactate levels were analyzed as logarithmic values.

Logistic regression including simple ED-available variables examined adjusted odds ratios with their 95% confidence intervals (CI) for complications, while controlling for potential confounders. Prior to model development, variables were assessed for collinearity, and no significant collinearity was detected. The final model, selected through backward elimination, included age, sex, hs-cTnT at arrival, initial lactate, final diagnosis of STEMI, Killip class, shock index (heart rate/systolic blood pressure), new ECG changes, and known comorbidities (heart failure, hypertension, hypercholesterolemia, diabetes mellitus, previous AMI, and coronary artery disease). Age, troponin, lactate, and shock index were treated as continuous variables in the analysis.

To compare the predictive accuracy of the different scores and the logistic regression model, the areas under the receiver operating characteristic (AUROC) curve were analyzed. Since a higher GRACE FFE score indicates a lower likelihood of an adverse event, its ROC curve was inverted to facilitate comparison with the other scores. The performance of the prediction tools was also evaluated at cutoff-values set to achieve a 90% sensitivity with all tools. Statistical significance was defined as *p* < 0.05 and all tests were two-sided.

Net reclassification improvement (NRI) analysis was used to further evaluate the predictive performance of the new logistic regression model compared to the known risk scores. For each comparator, continuous NRI with standardization was calculated. Reclassification was assessed separately for patients who experienced complications (events) and those who did not (non-events). Positive NRI values indicate improved reclassification by the new prediction model, whereas negative values indicate inferior reclassification by the new model. Overall NRI was calculated as the sum of the event and non-event components.

We used IBM SPSS Statistics 29 and R for all statistical analyses.

## 3 Results

**Fig. 1:**
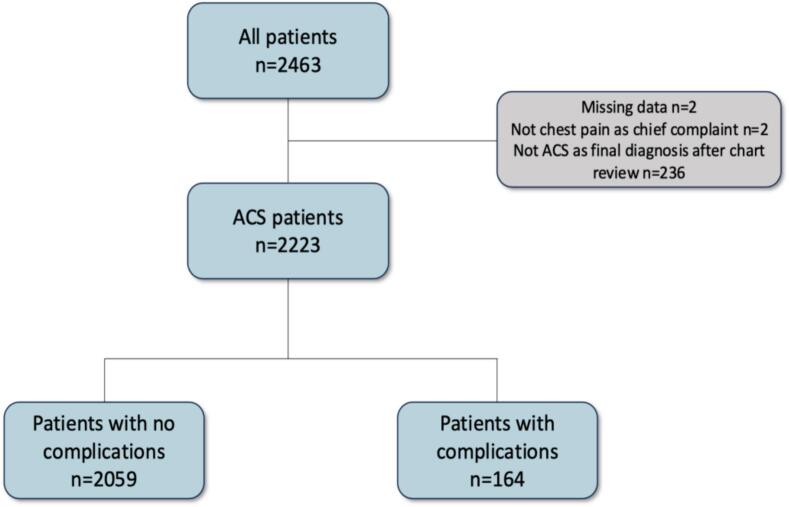
Patient flow chart.

**Fig. 2:**
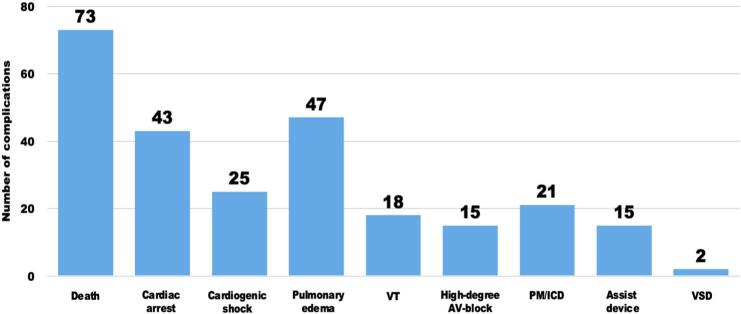
Types and numbers* of complications in ED ACS patients, *n* = 2223. *Some patients had more than one complication. VT – ventricular tachycardia; PM – pacemaker; ICD – implantable cardioverter-defibrillator; VSD – ventricular septum defect; Assist device - including intra-aortic balloon pump (IABP), percutaneous ventricular assist device (Impella pump) or extracorporeal membrane oxygenation (ECMO).

A total of 2463 ACS cases were identified via ICD-10 codes in the ESC-TROP database (Fig. 1) and subsequently underwent manual chart review. This identified 236 patients with no ACS, two patients with missing data, and two with a chief complaint other than chest pain, leaving 2223 patients in the final analysis. Of them, 164 had at least one complication, and many had multiple complications (Fig. 2). Death was the most common complication, followed by pulmonary edema. Nine patients had a procedural cardiac tamponade, and one had an iatrogenic aortic dissection, and these patients were not included in the complications group.

Table 1 shows the baseline patient characteristics. The mean age of all patients was 70 (range 32–100) years and 68% were male. Sixty-one percent of the patients had NSTEMI (type 1 or 2), 75% were admitted to the CCU, 85% underwent invasive procedures and 69% were revascularized. Among all ACS patients, 7.4% had at least one complication. Patients with complications were on average older (77.1 vs. 63.5 years) and more often female (40.2% vs 31.1%) compared to those without complications. Patients with complications also had a higher prevalence of comorbidities such as heart failure (18.9% vs. 7.7%), COPD (7.3% vs 3.8%), and dementia or mild cognitive impairment (11.5% vs. 3.1%). A Do-Not-Resuscitate (DNR) order was present in 43% of the patients with complications and in only 7% of those without complications. The corresponding percentages among patients ≥75 years were 64% versus 19%.

**Table 1:** Baseline patient characteristics.

	All patients n = 2223	Patients with complications *n* = 164	Patients with no complication *n* = 2059
Age, mean, SD	70.1 (12.7)	77.1 (14.1)	63.5 (12.4)
Age ≥ 75	830 (37.3%)	103 (62.8%)	727 (35.3%)
Sex, male	1516 (68.2%)	98 (59.8%)	1418 (68.9%)
Current smoker	398 (17.9%)	29 (17.7%)	369 (17.9%)
Family history of CAD	489 (22.0%)	15 (9.1%)	474 (23.0%)
BMI, mean, SD	27.5 (4.6)	26.3 (5.3)	27.6 (4.6)
DNR	216 (9.7%)	71 (43.3%)	145 (7.0%)

Previous diseases or interventions			
Hypertension	706 (31.8%)	63 (38.4%)	643 (31.2%)
CAD	594 (26.7%)	52 (31.7%)	542 (26.3%)
Previous AMI	413 (18.6%)	36 (22.0%)	377 (18.3%)
Chronic heart failure	190 (8.5%)	31 (18.9%)	159 (7.7%)
PAD	135 (6.1%)	11 (6.7%)	124 (6.0%)
Diabetes Mellitus	530 (23.8%)	41 (25.0%)	489 (23.7%)
COPD	90 (4.0%)	12 (7.3%)	78 (3.8%)
Hypercholesterolaemia	173 (7.8%)	7 (4.3%)	166 (8.1%)
Cerebrovascular disease	221 (9.9%)	19 (11.6%)	202 (9.8%)
Dementia	83 (3.7%)	19 (11.5%)	64 (3.1%)
Previous revascularization	292 (13.1%)	13 (7.9%)	279 (13.6%)
Previous PCI	264 (11.9%)	12 (7.3%)	252 (12.2%)
Previous CABG	41 (1.8%)	1 (0.6%)	40 (1.9%)
New ECG changes	1186 (53.4%)	133 (81.1%)	1053 (51.1%)

Treatment strategy			
Invasive	1880 (84.6%)	104 (63.4%)	1776 (86.3%)
Conservative	326 (14.7%)	52 (31.7%)	274 (13.3%)
Palliative	12 (0.5%)	8 (4.9%)	4 (0.2%)

Invasive procedures			
Coronary angiography index visit	1864 (83.9%)	103 (62.8%)	1761 (85.5%)
PCI index visit	1293 (58.2%)	72 (43.9%)	1221 (59.3%)
CABG index visit	225 (10.1%)	17 (10.4%)	208 (10.1%)

Admission ward			
CCU	1660 (74.7%)	105 (64.0%)	1555 (75.5%)
ICU	9 (0.4%)	8 (4.9%)	1 (0.0%)
Cardiology ward	77 (3.5%)	5 (3.0%)	72 (3.5%)
Telemetry ward	368 (16.6%)	22 (13.4%)	346 (16.8%)
Medical ward	93 (4.2%)	21 (12.8%)	72 (3.5%)
ED	16 (0.7%)	3 (1.8%)	13 (0.6%)

Final diagnosis			
STEMI	339 (15.2%)	71 (43.3%)	268 (13.0%)
NSTEMI	1268 (57.0%)	77 (47.0%)	1191 (57.8%)
UA	527 (23.7%)	6 (3.7%)	521 (25.3%)
Type 2 infarction	89 (4.0%)	10 (6.1%)	79 (3.8%)

Blood tests			
Hemoglobin, mean, SD	140 (18)	133 (20)	141 (18)
hs-cTnT at arrival, median, IQR	39 (104)	110 (435)	37 (97)
Lactate at arrival, median, IQR	1.4 (1)	2.1 (2)	1.4 (1)
Glucose, median, IQR	6.9 (3)	8.8 (5)	6.8 (3)
Creatinine, median, IQR	83 (29)	93 (54)	82 (27)

CAD – coronary artery disease; AMI – acute myocardial infarction; PAD – peripheral artery disease; COPD – chronic obstructive pulmonary disease; DNR – Do-not-resuscitate; PCI – percutaneous coronary intervention; CABG – coronary artery bypass graft surgery; STEMI – ST-elevation myocardial infarction; NSTEMI – Non-ST elevation myocardial infarction; UA – unstable angina; hs-cTnT – high-sensitivity cardiac troponin T; CCU – cardiac care unit; ICU – intensive care unit; ED – emergency department;

Patients with complications were less often treated invasively (63.4% vs. 86.3%), revascularized (54.3% vs 69.4%), or admitted to the CCU (64.0% vs. 75.5%). Blood tests showed higher levels of hs-cTnT, lactate, glucose, and creatinine in those with complications. Additionally, new ischemic changes on the arrival ECG were more frequent in this group (81.1% vs. 51.1%).

### 3.1 Logistic regression

Table 2 presents the results from the univariable and multivariable logistic regression analyses. After excluding patients with complications at the ED (*n* = 45) and patients with palliative care from admission (*n* = 8), as well as those with missing data, 2057 patients were included in the analysis. Of them, 108 (5.3%) had at least one complication. After adjusting for sex and comorbidities, the following factors were significant predictors of complications: Age (OR 1.04, 95% CI 1.02–1.06), STEMI (OR 2.02, 95% CI 1.21–3.38), troponin at arrival (OR 1.80, 95% CI 1.28–2.52), initial lactate (OR 11.62, 95% CI 3.97–33.97), shock index (OR 3.85, 95% CI 1.40–10.57), Killip class at arrival (OR 2.24, 95% CI 1.31–3.81 for Killip class II and OR 6.48, 95% CI 2.06–20.40 for Killip class III), and new ECG changes (OR 2.11, 95% CI 1.22–3.63).

**Table 2:** Univariable and multivariable logistic regression analyses for the prediction of complications in 2057 ACS patients at the ED.

		Univariable logistic regression		Multivariable logistic regression^a^, ^b^	
	n	OR (95% CI)	*p*-value	OR (95% CI)	p-value
STEMI					
No	1762	Ref.		Ref.	
Yes	295	3.74 (2.47–5.66)	<0.001	2.02 (1.21–3.38)	0.007
Killip class			<0.001		<0.001
1	1845	Ref.		Ref.	
2	196	4.58 (2.90–7.23)	<0.001	2.24 (1.31–3.81)	0.003
≥ 3	16	25.36 (9.25–69.53)	<0.001	6.48 (2.06–20.40)	0.001
Shock Index	2057	20.45 (8.86–47.18)	<0.001	3.85 (1.40–10.57)	0.009
Age	2057	1.07 (1.05–1.08)	<0.001	1.04 (1.02–1.06)	<0.001
Hs-cTnT Lg	2057	3.33 (2.50–4.44)	<0.001	1.80 (1.28–2.52)	<0.001
Lactate Lg	2057	41.25 (16.26–104.68)	<0.001	11.62 (3.97–33.97)	<0.001
Known heart failure					
No	1894	Ref.		Ref.	
Yes	163	2.87 (1.72–4.80)	<0.001	1.67 (0.86–3.22)	0.128
New ECG changes					
No	970	Ref.		Ref.	
Yes	1087	3.49 (2.18–5.58)	<0.001	2.11 (1.22–3.63)	0.007
Sex					
Male	1416	Ref.		Ref.	
Female	641	1.62 (1.09–2.41)	0.016	1.03 (0.65–1.62)	0.908
Comorbidities^c^	2057	1.09 (0.96–1.25)	0.197	0.99 (0.83–1.18)	0.927

STEMI – ST-elevation myocardial infarction; Hs-cTnT – high-sensitivity cardiac troponin T; Lg – logarithm; ECG – electrocardiogram;

a Adjusted for sex and comorbidity.

b Hosmer and Lemeshow Test, *p* = 0.649.

c Including hypertension, hypercholesterolemia,diabetes mellitus, known coronary artery disease and previous myocardial infarction.

### 3.2 Prediction of complications

**Fig. 3:**
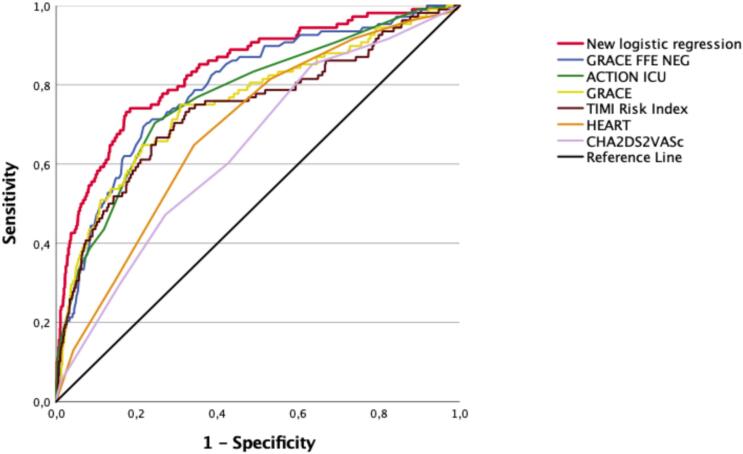
ROC curves of published risk scores and a new logistic regression model for the prediction of complications in ACS patients, *n* = 2056.

The distributions of the risk score points in the cohort are presented in Supplement Fig. 1. ROC curves for all scores and the logistic regression model in the 2056 patients are shown in Fig. 3, and AUROCs in Table 3. GRACE FFE (AUROC 0.79, 95% CI 0.75–0.84), ACTION ICU (0.77, 95% CI 0.72–0.82), GRACE (0.76, 95% CI 0.70–0.81), and TIMI (0.74, 95% CI 0.68–0.79) performed equally well and were acceptable predictors of complications, whereas the HEART (0.69, 95% CI 0.64–0.74) and CHA₂DS₂-VASc (0.64, 95% CI 0.59–0.69) scores performed poorly. The logistic regression model had excellent discrimination and the highest AUROC (0.84, 95% CI 0.80–0.88). To facilitate comparison across all risk scores, diagnostic performances with cut-offs set to yield a 90% sensitivity are presented in Table 4. With these cut-offs, the specificities of the scores ranged from 18% to 43%, which was clearly below the 52% specificity of the logistic regression model.

NRI analysis demonstrated positive overall values for the new logistic regression model compared with all six published risk scores (Table 5). These improvements were predominantly driven by substantial positive non-event NRI components, indicating more accurate classification of patients who did not develop complications. In contrast, event- specific NRI components were negative for the logistic regression model compared with all scores, reflecting limited upward reclassification of patients with complications.

**Table 3:** Area under the ROC curve (AUROC) of the studied risk scores and the new logistic regression model.

Score	AUROC	Std Error	p-value	95% CI
Logistic regression model	0.837	0.021	0.000	0.796–0.879
GRACE FFE (neg)	0.792	0.023	0.000	0.748–0.837
ACTION ICU	0.772	0.025	0.000	0.724–0.820
GRACE	0.756	0.027	0.000	0.703–0.809
TIMI	0.739	0.028	0.000	0.683–0.794
HEART	0.687	0.025	0.000	0.638–0.736
CHA₂DS₂-VASc	0.640	0.027	0.000	0.588–0.692

CI – confidence interval.

**Table 4:** Cut-offs and accuracy of the risk prediction tools for complications in ED ACS patients, n = 2056.

Variable	Cut-off	Complication					
		Yes	No	Sensitivity	Specificity	PPV	NPV
New logistic regression							
	≥ 0.022 (Positive)	98	941				
	< 0.022 (Negative)	10	1007	90.7%	51.7%	9.4%	99.0%
GRACE FFE (NEG)							
	≥ −260.5 (Positive)	98	1103				
	< −260.5 (Negative)	10	845	90.7%	43.4%	8.2%	98.8%
ACTION ICU							
	≥ 2.5 (Positive)	98	1349				
	< 2.5 (Negative)	10	599	90.7%	30.7%	6.8%	98.4%
GRACE							
	≥ 80.8 (Positive)	98	1472				
	< 80.8 (Negative)	10	476	90.7%	24.4%	6.2%	97.9%
TIMI Risk Index							
	≥ 16.6 (Positive)	98	1533				
	< 16.6 (Negative)	10	415	90.7%	21.3%	6.0%	97.6%
HEART							
	≥ 5.5 (Positive)	99	1436				
	< 5.5 (Negative)	9	512	91.7%	26.3%	6.4%	98.3%
CHA₂DS₂-VASc							
	≥ 0.5 (Positive)	99	1606				
	< 0.5 (Negative)	9	342	91.7%	17.6%	5.8%	97.4%

NPV – negative predictive value.

PPV – positive predictive value.

**Table 5:** Net Reclassification Improvement analysis for the comparison of our logistic regression model with 6 published risk scores.

Reclassification	NRI		
	All	Event	Non-event
ACTION ICU score	0.45 (0.36–0.54)	−0.43 (−0.52; −0.34)	0.88 (0.86; 0.89)
GRACE FFE score	0.49 (0.41; 0.57)	−0.093 (−0.17; −0.017)	0.58 (0.56; 0.60)
GRACE score	0.36 (0.26; 0.45)	−0.56 (−0.66; −0.47)	0.92 (0.91; 0.93)
TIMI risk index score	0.47 (0.38; 0.57)	−0.40 (−0.49; −0.31)	0.87 (0.86; 0.89)
HEART score	0.097 (0.034; 0.17)	−0.86 (−0.92; −0.79)	0.96 (0.95; 0.97)
CHA2DS2VASc score	0.44 (0.34; 0.53)	−0.16 (−0.25; −0.064)	0.60 (0.57; 0.62)

## 4 Discussion

In the present study, we found that only about 7% of all patients with ACS suffered a complication within 30 days, and that the most common one was death. The previously published risk scores were only acceptable or poor predictors of complications. A logistic regression model based on readily available information in the ED had excellent discrimination and was the best predictor of complications in the present ACS population.

Among the variables in our model, age is a well-known risk factor in ACS patients [21], and 63% of the patients with complications in our cohort were ≥ 75 years. Of all patients with complications, a DNR order was present at admission for 43% (for 64% of those ≥75 years), suggesting limited life expectancy. Some deaths in the elderly were even expected, justifying a non-invasive strategy and admission to a ward with lower-level care. Consensus in the literature is lacking regarding the benefits of invasive treatment in the elderly. Some authors report a benefit [22] and others do not [23], [24], the latter arguing that each patient should receive an individual treatment plan. Based on the present results, individualized plans for elderly ACS patients seem reasonable, without routine CCU admission or coronary angiography.

Vital signs such as heart rate and systolic blood pressure are known to be important predictors of outcome in ACS patients, and these variables are also included in the GRACE, GRACE FFE, and ACTION ICU models [7], [8]. The TIMI risk index is based solely on heart rate, systolic blood pressure, and age, and is a strong predictor of in-hospital mortality [15]. A high Killip class is a recognized predictor of bad outcomes in STEMI patients associated with increased in-hospital mortality and a higher risk of heart failure-related hospitalizations [25]. The present results thus confirm that vital signs and Killip class are significant predictors of complications in ACS patients at the ED.

Our model also included lactate, a known predictor of mortality in critically ill patients [26], [27]. We excluded patients with hemodynamic instability (or any complication) before ward admission, and our data therefore indicate that higher lactate levels are associated with a higher risk of complications even in initially stable ED ACS patients.

The type and size of infarction also appear to be important in the prediction of complications. Patients with STEMI and larger infarctions (higher initial hs-cTnT levels) were more likely to suffer complications in our study population, and this is in line with previous studies [28], [29]. Our results support the notion that stable patients with non-ST-elevation ACS and a low hs-cTnT should be considered low risk, and that admission to a ward with lower-level monitoring might be an option in these patients.

Even though many of the variables in our logistic regression model are present in the studied risk scores (e.g. age, blood pressure, heart rate, hs-cTnT, ECG changes), the scores performed worse and were at best acceptable predictors. This might not be surprising since most scores were originally not designed to predict 30-day complications as in this study. For example, GRACE was developed to estimate mortality up to six months [7], ACTION ICU was developed for NSTEMI patients, and GRACE FFE for NSTEMI and UA patients [30].

The HEART score is a widely used tool in ED chest pain patients, designed to predict the short-term risk of ACS and MACE [31]. It is easy to calculate and interpret. However, its prediction of complications in the present study was only acceptable. The GRACE FFE performed best of the published scores, but it is complex to calculate and interpret which makes it unpractical for daily use. The GRACE score, though mentioned in guidelines, performed worse than GRACE FFE and is similarly complex to calculate.

We compared the performance of the risk prediction tools at cut-off values set to identify 90% of the complications, if possible. At these cut-offs, all scores achieved 90% sensitivity but displayed very low specificity. For instance, CHA₂DS₂-VASc score reached 90% sensitivity at the cut-off 1 point, which would put all women in the high-risk group. Our logistic regression model had the highest specificity and was able to correctly rule out complications in 49% of all patients (Table 4). Our model thereby has the potential to help the ED physician to safely spare a significant number of CCU/ICU admissions, saving the cardiologists' time and resources for true high-risk patients.

Consistent with these findings, the NRI analyses indicated that the primary strength of the logistic regression model lies in improved identification of patients at low risk for complications rather than enhanced detection of events. This pattern is clinically relevant in the ED setting, where reducing false-positive risk classification can limit unnecessary monitoring, and resource utilization. The negative event-specific NRI components observed across comparator scores highlight the sensitivity of continuous NRI to directional changes in predicted risk rather than absolute risk stratification and reinforce the need to interpret NRI alongside complementary performance measures, including discrimination, calibration, and clinically meaningful operating characteristics, rather than as a standalone indicator of model performance.

Future work should explore the use of machine learning and artificial intelligence (AI) models, including approaches that incorporate clustering, to achieve more refined risk stratification and to evaluate their utility in the ED setting.

## 5 Strengths and limitations

This was an observational study that used data from the ESC TROP trial. The patients were consecutive and included at five different EDs at hospitals with different levels of care – two university hospitals, two community hospitals, and one rural hospital – with varying monitoring capabilities. This diversity reduces the likelihood that systematic differences in surveillance or documentation would bias the results and supports the generalizability of our findings across different hospital settings.

Further, we compared our logistic regression model with six published risk scores, which strengthens the internal validity of the study. The study used data from registries as well as from manual chart review. Most charts were reviewed by two authors but one of the authors (TN) reviewed all charts, which should ensure a homogenous collection of information in all patients. While retrospective reviews risk bias from missing data and the treating physician's interpretations, incorporating objective test results likely reduced this risk.

Information regarding some risk factors (family history and smoking status) were occasionally missing, especially among the elderly, potentially affecting scores. However, most data were available, and we believe that our assumptions introduced minimal bias. Further, family history was likely less relevant in older patients.

A low LVEF on echocardiography is associated with increased mortality in ACS patients and studies suggest that LVEF assessment is beneficial in risk prediction [32], [33]. Due to the low number of echocardiograms performed at the ED, we were unable to include LVEF in our regression analysis. Prospective studies including LVEF assessment at the ED seem warranted.

ECG interpretation by a single author (TN) ensured consistency but is a limitation. Computerized ECG interpretation might have altered the results and could be included in future machine learning models for prediction of ACS complications.

In region Skåne, STEMI patients are often identified in the ambulance and admitted directly to the CCU, bypassing the ED. The number of STEMI patients in our ED population was therefore low, and it seems likely that the complication rates would be different if all STEMI patients were included. The results should therefore be interpreted with caution in health care systems with a different organization of STEMI patient care.

## 6 Conclusion

In this study, 7% of all ED ACS patients suffered at least one serious complication. In the prediction of these complications, a logistic regression model using simple variables available at the ED had excellent discrimination and outperformed six published risk scores. Improved risk assessment in ED ACS patients may allow more effective use of cardiac monitoring and hospital resources.

## Funding sources/disclosure

The study was supported by an ALF research grant at 10.13039/501100011077Skåne University Hospital, by a grant from 10.13039/501100009780Region Skåne, and by a grant from the 10.13039/501100003793Swedish Heart-Lung foundation. This study was part of the AIR Lund (Artificially Intelligent use of Registers at Lund University) research environment and received funding from the Swedish Research Council (VR; grant no. 2019-00198). UE was the recipient of all grants. There was no industry involvement. Funding organizations had no role in the planning, design, or conduct of the study, collection, analysis or interpretation of data, or preparation, review, or approval of the manuscript. None of the authors report any conflicts of interest.

## CRediT authorship contribution statement

**T. Nilsson:** Writing – review & editing, Writing – original draft, Visualization, Validation, Project administration, Methodology, Investigation, Formal analysis, Data curation, Conceptualization. **M. Strömfors:** Writing – review & editing, Writing – original draft, Investigation, Data curation. **A. Trägårdh:** Writing – review & editing, Writing – original draft, Investigation, Data curation. **A. Mokhtari:** Writing – review & editing, Writing – original draft, Validation, Supervision, Methodology, Data curation. **A.M. Khoshnood:** Writing – review & editing, Validation, Supervision. **U. Ekelund:** Writing – review & editing, Visualization, Validation, Supervision, Software, Resources, Project administration, Methodology, Investigation, Funding acquisition, Data curation, Conceptualization.

## Ethical statement

The authors of this manuscript confirm that it is their own original work and is not currently under consideration for publication elsewhere.

The paper presents original research and analysis truthfully and in its entirety.

The article's publication is approved by all authors. The study was approved by the regional ethics review board in Lund, Sweden.

## Declaration of competing interest

The authors declare that they have no known competing financial interests or personal relationships that could have appeared to influence the work reported in this paper.

## Appendix A Supplementary data

Supplement Fig. 1 Risk score distributions in all ACS patients. Supplement Fig. 1
