# Supplementary material for: Predicting complications in emergency department patients with acute coronary syndrome – Existing risk scores versus a new logistic regression model
Source: Am Heart J Plus. 2026 Feb 10;63:100736. doi: 10.1016/j.ahjo.2026.100736 (PMC12925123; doi:10.1016/j.ahjo.2026.100736)
Supplement: Supplement Fig. 1 — Risk score distributions in all ACS patients. [file mmc1.docx]

**Supplement Figure 1. Risk score distributions in all ACS patients**

1. **HEART score, n=2220***


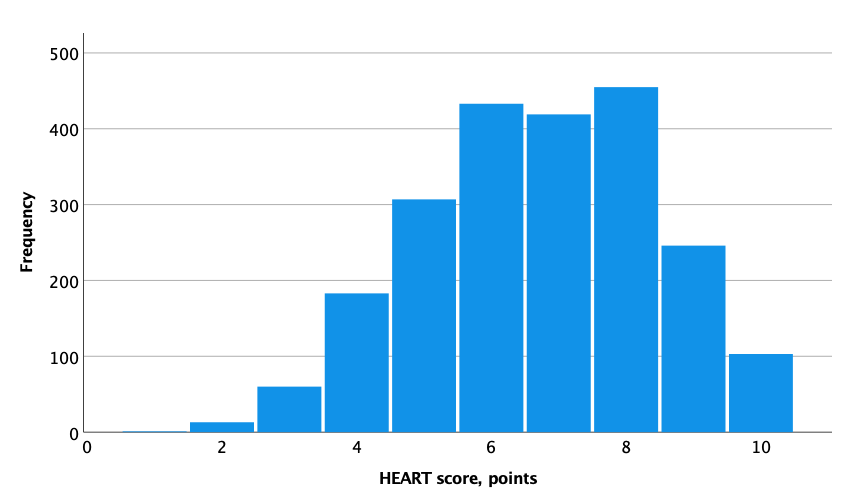


*missing n=3

1. **ACTION ICU score, n=2220***


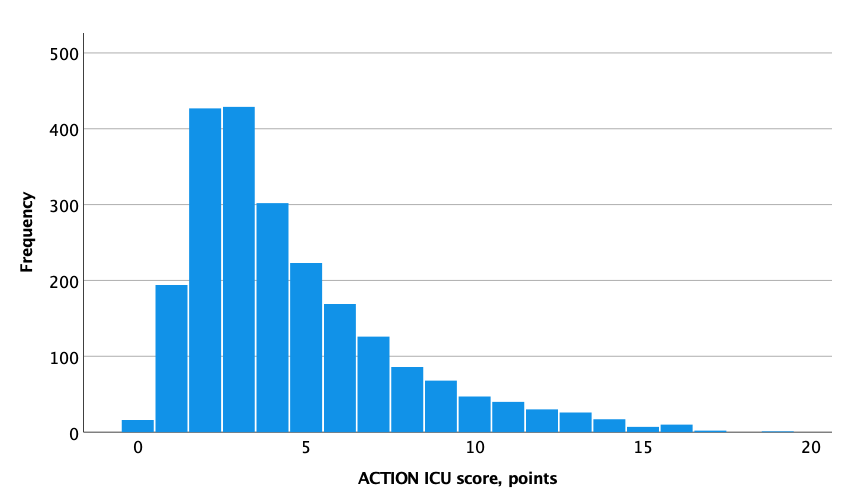


*missing n=3

1. **CHA2DS2VASc score, n=2220***


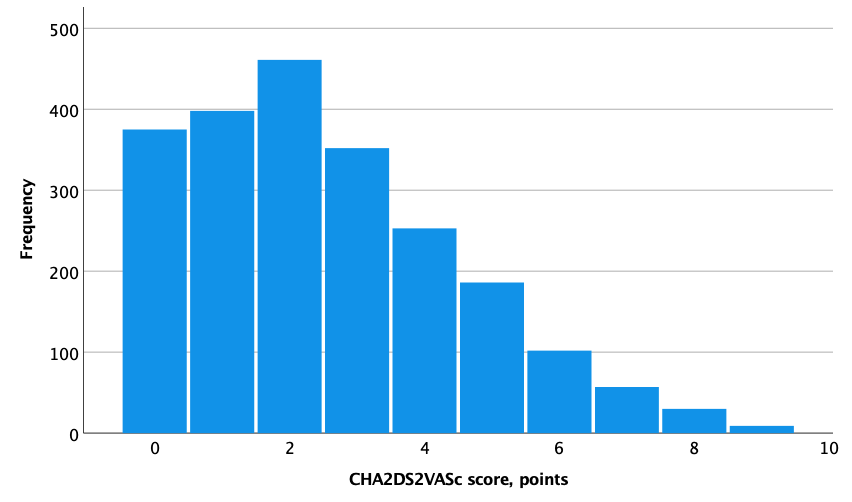


*missing n=3

1. **GRACE score, n=2222***


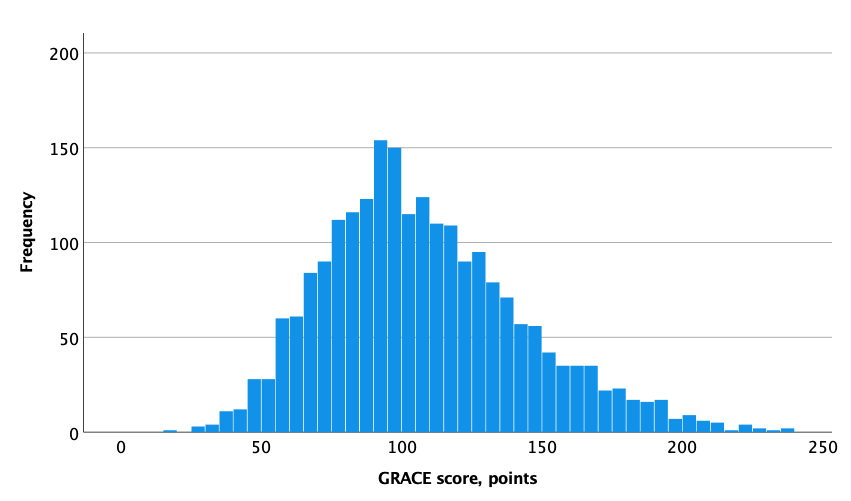


*missing n=1

1. **GRACE FFE score, n=2220***


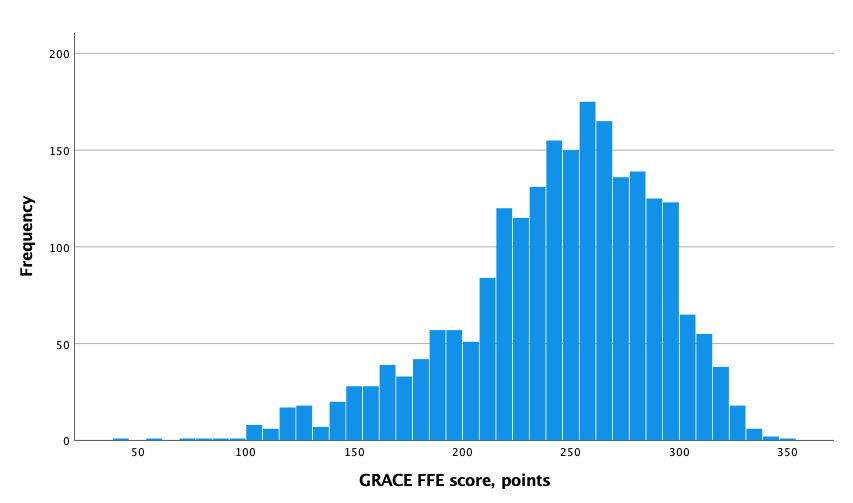


*missing n=3

1. **TIMI Risk Index score, n=2221***


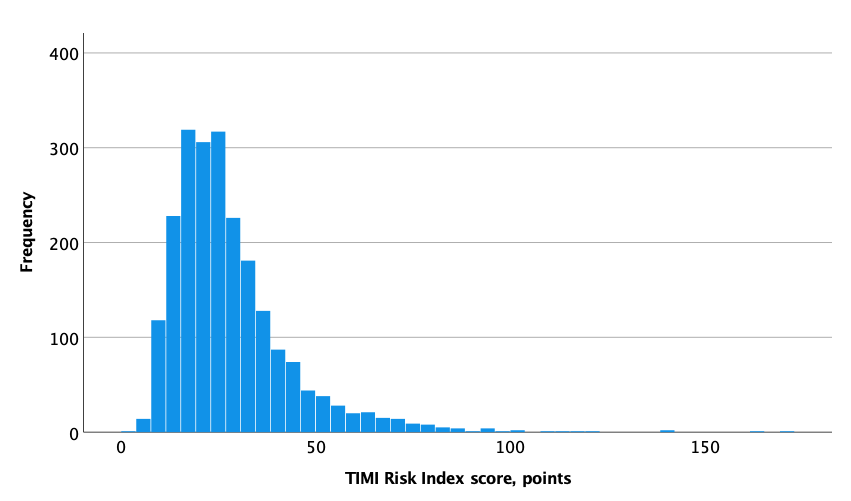


*missing n=2
